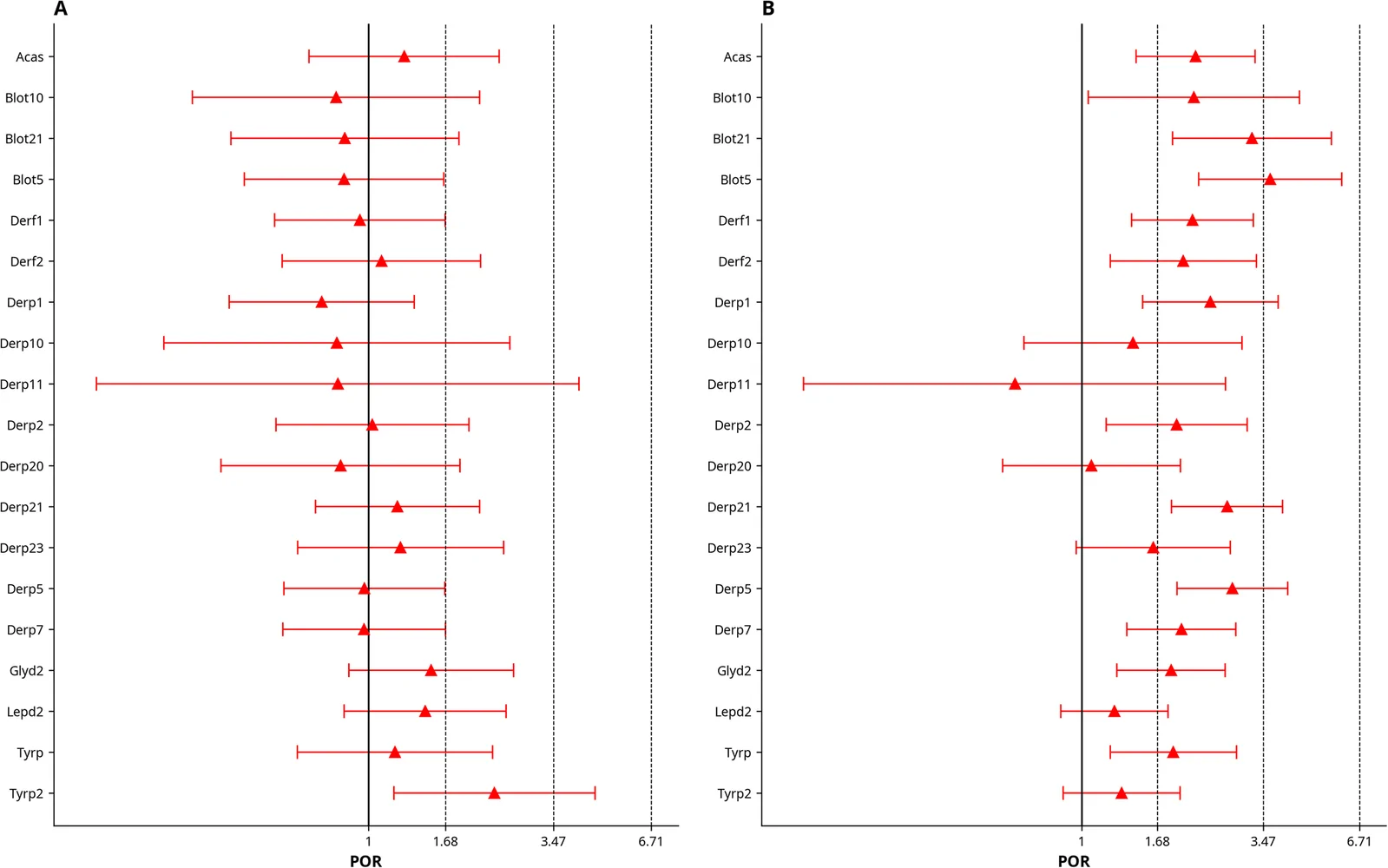# Regional diversity of major house dust mite allergens in Spain: Molecular profiles and clinical correlates

**DOI:** 10.1016/j.jacig.2026.100785

**Published:** 2026-08-25

**Authors:** Manuel J. Rial, David El-Qutob, Javier Alcover, Laura Villamayor, Montserrat Martínez-Gomariz, Ruperto González-Pérez

**Affiliations:** a Allergy Department. Complexo Hospitalario Universitario de Ferrol, A Coruña, Spain; b Allergy Service, University Hospital de la Plana, Castellón, Spain; c Diater Laboratorios, Madrid, Spain; d Allergy Service, Hospital Universitario de Canarias, San Cristóbal de La Laguna, Tenerife, Spain

**Keywords:** Dust mites, component-resolved diagnosis, Dermatophagoides, Lepidoglyphus, Blomia

## Abstract

**Background:**

House dust mites (HDMs) are a major cause of respiratory allergies worldwide. Molecular diagnosis has allowed a more precise characterization of sensitization patterns, which may have clinical and therapeutic relevance. However, large-scale data on molecular HDM sensitization profiles in real-world settings remain limited in Spain.

**Objective:**

We aimed to determine the seroprevalence and major allergens of HDM molecular components across different Spanish regions and to explore their association with allergic phenotypes, including allergic rhinitis (AR) and asthma.

**Methods:**

A multicenter cross-sectional study was conducted in 5 Spanish regions. A total of 458 serum samples from patients with suspected HDM allergy were analyzed using a multiplex molecular diagnostic platform. Sensitization was defined as specific IgE ≥ 0.3 kUA/L. Allergens with a prevalence ≥50% were considered major allergens. Associations with AR and asthma were evaluated by prevalence odds ratios (PORs) and chi-square tests.

**Results:**

The most prevalent HDM allergens were Der p 23 (85.2%), Der f 2 (82.3%), Der p 2 (81.0%), Der p 1 (77.5%), and Der f 1 (71.0%). Regional differences were observed, with Galicia showing high sensitization to Gly d 2 and Lep d 2 (both 57%). In the Canary Islands, >50% of patients were sensitized to Gly d 2, Lep d 2, Der p 21, Der p 5, Der p 7, and Blot 5. Sensitization to Blot 5 showed a moderate association with asthma (POR, 3.64), whereas Tyr p 2 was linked to persistent AR (POR, 2.33). Sensitization to Lep d 2 had variable associations with AR severity across regions.

**Conclusions:**

Der p 23, Der f 2, Der p 2, Der p 1, and Der f 1 are the major HDM allergens in Spain, with regional variability suggesting environmental or genetic modifiers. Certain molecular allergens are associated with more severe or persistent allergic phenotypes, highlighting the value of component-resolved diagnosis for risk stratification and immunotherapy planning.

House dust mites (HDMs) are among the most prevalent sources of indoor allergens and play a central role in the development of allergic rhinitis (AR) and asthma worldwide.^1^ Traditionally, diagnostic strategies have relied on extract-based testing, which does not distinguish between individual allergenic components. The introduction of component-resolved diagnostics (CRD) has enabled a more precise characterization of sensitization patterns at the molecular level, offering improved diagnostic accuracy, phenotypic stratification, and better guidance for allergen immunotherapy (AIT).^2^^,^^3^

Spain presents a unique setting to study HDM allergy, as many of its regions—particularly Galicia, the Basque Country, Valencia, the Balearic Islands, and the Canary Islands—are coastal or insular areas with high relative humidity throughout the year. These climatic conditions create an environment of sustained high mite exposure, which contributes to a heavy allergenic burden. Despite this, molecular-level data on HDM sensitization patterns in Spanish patients remain scarce.

This multicenter study aims to determine the seroprevalence and molecular profile of dust mite allergen sensitization across different Spanish regions with high environmental mite exposure. By including diverse geographic areas, this study provides a comprehensive view of regional variability and contributes to the definition of a more precise molecular sensitization profile in the Spanish population. Associations with clinical phenotypes such as AR and asthma are also explored to assess the potential diagnostic and prognostic value of individual allergenic components.

## Methods

### Study design and population

This was a multicenter, cross-sectional observational study conducted across 5 Spanish regions with high environmental HDM exposure: Galicia, the Basque Country, Valencia, the Balearic Islands, and the Canary Islands. A total of 458 patients were recruited from first-time allergy consultations in participating hospitals and outpatient clinics. AR persistence and severity were classified according to the Allergic Rhinitis and its Impact on Asthma guidelines. Asthma diagnosis and severity classification were performed following the Global Initiative for Asthma recommendations.^4^^,^^5^

### Inclusion criteria

• First-time visit to an allergy department.
• Presence of clinical symptoms suggestive of HDM allergy, including AR, conjunctivitis, asthma, and/or atopic dermatitis.
• Confirmed sensitization to HDM extracts by skin prick test.

### Exclusion criteria

• Patients for whom skin prick tests are contraindicated.
• Patients who are or have been treated with specific immunotherapy against dust mites.
• Patients undergoing treatment with drugs that interfere with the immune system.
• Lack of cooperation or compliance.
• Having lived in the region for less than 3 years.
• Onset of symptoms prior to settling in the region.

### Molecular diagnostic platform

Specific IgE sensitization was assessed using the ALEX^2^ (Allergy Explorer 2) multiplex in vitro assay (MacroArray Diagnostics, Vienna, Austria). ALEX^2^ is a nanotechnology-based immunoassay that simultaneously quantifies IgE antibodies against a wide array of allergens, including whole extracts and molecular components. For this study, 19 HDM molecular allergens were selected, representing multiple species and protein families (group 1, 2, 5, 7, 10, 11, 21, and 23).

Sensitization to a given component was defined as specific IgE ≥ 0.3 kUA/L, as recommended by the manufacturer. Allergens were considered major when their prevalence in the total sample was ≥50%.

### Ethical considerations

The study protocol was reviewed and approved by the corresponding local ethics committees in each participating region. Written informed consent was obtained from all patients prior to inclusion. The study was conducted in accordance with the ethical principles of the Declaration of Helsinki and applicable regulatory requirements for clinical research involving human participants.^6^

### Statistical analysis

Descriptive statistics were used to summarize sociodemographic variables and allergen sensitization rates. Regional differences were evaluated using chi-square or Fisher exact tests. Because this is a cross-sectional study, associations between sensitization and clinical outcomes (AR and asthma phenotypes) were assessed using prevalence odds ratios (PORs) with 95% confidence intervals (CIs), as derived from 2 × 2 contingency tables. Multivariable adjustment was not performed because the available dataset consisted of aggregated outputs from the statistical report; this limitation is explicitly acknowledged. A *P* value < .05 was considered statistically significant.

## Results

### Patient characteristics

A total of 458 patients were included in the study. The majority were adults (84.3%), with a balanced gender distribution (52.2% female). Patients were recruited from Galicia (158), the Basque Country (97), Valencia (50), the Balearic Islands (100), and the Canary Islands (53). Most patients resided in urban areas (78.6%), although Galicia showed a significantly higher proportion of rural or semi-rural dwellings (33.5%, *P <* .004).

A recruitment flowchart summarizing patient inclusion and analysis is provided in Fig E1 (in the Online Repository at www.jaci-global.org).

### Prevalence of AR and asthma

AR was present in 90.2% of patients, predominantly in a persistent form (77.8%). Asthma was diagnosed in 48.5%, with significant regional differences: in the Canary Islands, asthma prevalence reached 88.7% (*P <* .001). In Valencia, asthma was less frequent (28%, *P* = .006), with predominance of intermittent asthma. The co-occurrence of AR and asthma varied across regions, being most common in the Balearic and Canary Islands (>60%) (Table I).

**Table I:** Demographic characteristics and prevalence of allergic rhinitis and asthma among patients included in the study across the 5 Spanish regions∗

Variable	Total (N = 458)	Galicia (n = 158)	Basque Country (n = 97)	Valencia (n = 50)	Balearic Islands (n = 100)	Canary Islands (n = 53)	*P* value
Age distribution							<.001
<18 y	72 (15.7%)	22 (13.9%)	0	0	50 (50.0%)	0	
18-40 y	170 (37.1%)	64 (40.5%)	32 (33.0%)	24 (48.0%)	29 (29.0%)	21 (39.6%)	
41-60 y	129 (28.2%)	42 (26.6%)	39 (40.2%)	16 (32.0%)	6 (6.0%)	26 (49.1%)	
>60 y	87 (19.0%)	30 (19.0%)	26 (26.8%)	10 (20.0%)	15 (15.0%)	6 (11.3%)	
Sex							.12
Female	239 (52.2%)	70 (44.3%)	56 (57.7%)	27 (54.0%)	53 (53.0%)	33 (62.3%)	
Male	219 (47.8%)	88 (55.7%)	41 (42.3%)	23 (46.0%)	47 (47.0%)	20 (37.7%)	
Allergic rhinitis	413 (90.2%)	155 (98.1%)	97 (100%)	50 (100%)	100 (100%)	11 (20.8%)	<.001
Asthma	222 (48.5%)	63 (39.9%)	38 (39.2%)	14 (28.0%)	60 (60.0%)	47 (88.7%)	<.001

∗ Participant characteristics by region. *P* values were calculated using chi-square tests comparing proportions across regions.

### Major allergen sensitization

The following allergens were classified as major (≥50% sensitization rate in the full cohort): Der p 23 (85.2%), Der f 2 (82.3%), Der p 2 (81.0%), Der p 1 (77.5%), and Der f 1 (71.0%). In second place, we find the allergens Lep d 2 (48.9%), Gly d 2 (47.6%), Der p 5 (46.9%), Der p 7 (45.9%), and Der p 21 (44.8%). These allergens cannot be considered major allergens in this population sample. It should also be noted that the allergens Blot 10, Der p 10, and Der p 11 have prevalences lower than 10% and, in the case of Der p 11, its prevalence is lower than 5% (Fig 1).

**Fig 1:**
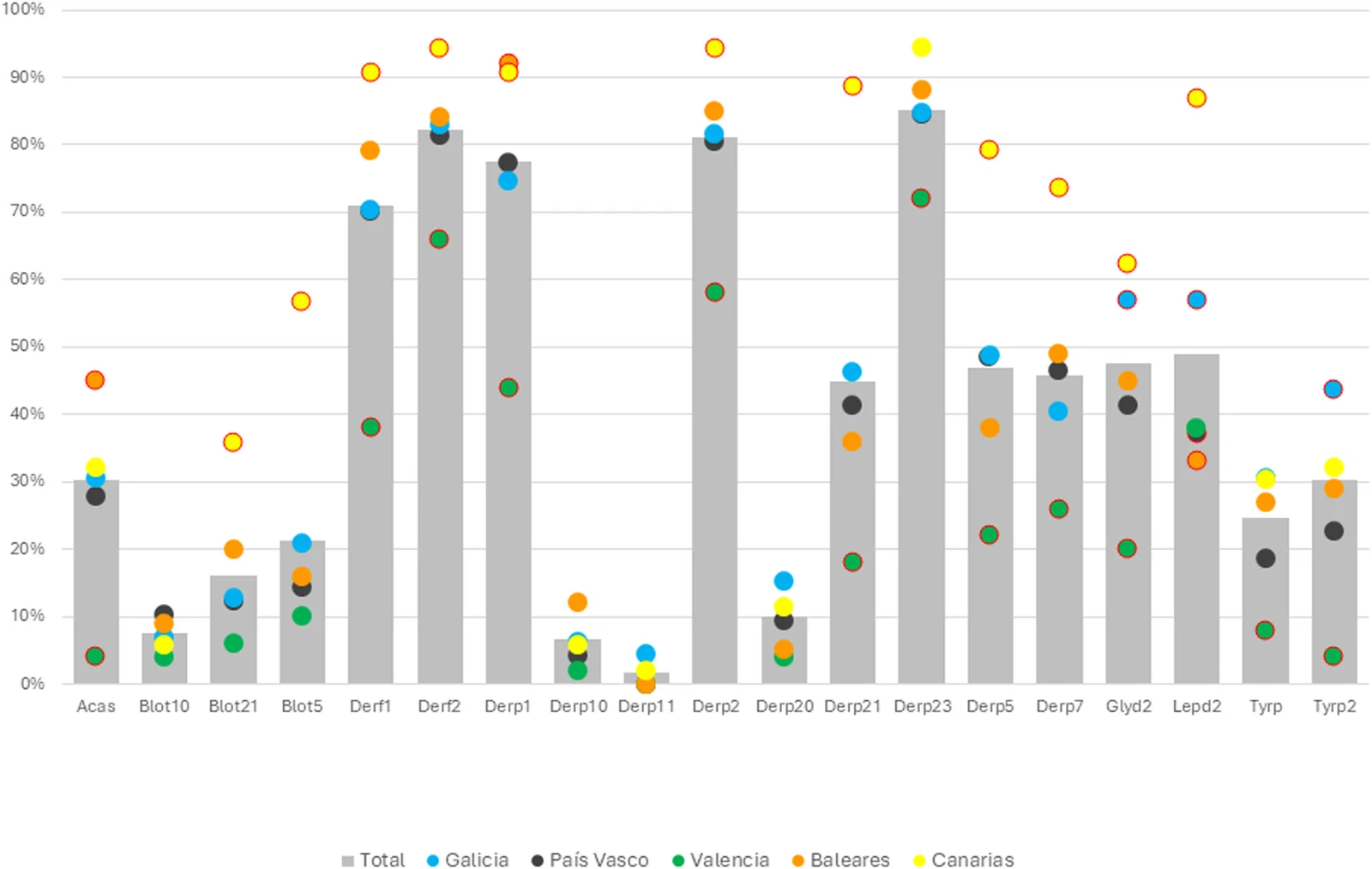
Prevalence by regions in Spain. The *red circle* shows samples with a statistically significant difference with respect to the total, according to the chi-square test with a confidence level of 95%.

These were consistently dominant across most regions, but with statistically significant variations with respect to the total population: In Galicia elevated sensitization to Gly d 2 and Lep d 2 (both 57%) were detected, making them regional major allergens. In the Canary Islands, additional major allergens included Der p 21 (88.7%), Der p 5 (79.2%), Der p 7 (73.6%), Blot 5 (56.6%), Lep d 2 (86.8%), and Gly d 2 (62.3%). In the Balearic Islands, a high prevalence of Der p 1 (92%) and Aca s (45%) was detected. Valencia showed significantly lower sensitization rates across most allergens.

### Allergen combinations

More than half of the cohort (52%) were sensitized to between 6 and 11 allergens. Approximately 24.9% presented sensitivity to combinations between 1 and 5 allergens and 19% to combinations between 12 and 17 allergens (Fig 2, *A*).

**Fig 2:**
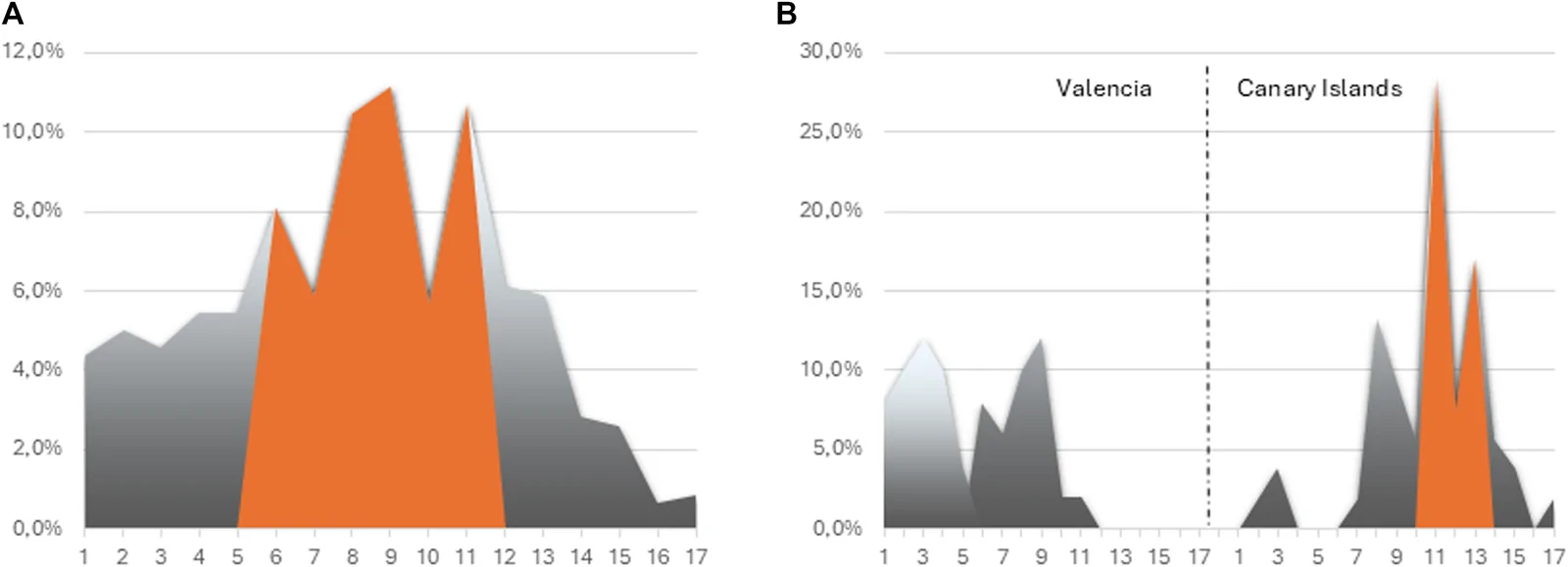
Number of allergens per combination in the total sample **(A)** and details of the population in Valencia and the Canary Islands **(B)**.

This distribution was similar across most regions; however, clear differences were observed in Valencia and the Canary Islands (Fig 2, *B*). In Valencia, there are no combinations with more than 11 allergens; 44% of the sample presents combinations between 1 and 5 allergens; and 40% between 6 and 11. It should be remembered that this community presents a significantly higher percentage of patients with no sensitivity to any allergen; as well as patients sensitive to 3 allergens.

With regard to the Canary Islands, 52.8% of patients present combinations between 11 and 13 allergens, this percentage being significantly higher than in the other communities. Furthermore, it should be noted that 86.8% of the patients present combinations between 8 and 14 allergens; a very small range compared with the average distribution and showing little sensitivity to combinations with a small number of allergens.

### Patterns of allergen cosensitization and clinical associations

Analysis of sensitization patterns revealed that cosensitization to multiple HDM allergens was the rule rather than the exception. The most frequent binary combination was Der f 2 and Der p 2, observed in more than 80% of patients. When a third component was considered, combinations involving Der f 2, Der p 2, and Der p 1 or Der p 23 were also highly prevalent, each affecting over 75% of the cohort. Regional variations were noted: in Galicia, 68.9% of patients exhibited patterns including Gly d 2 and/or Lep d 2, whereas in the Canary Islands, 94.6% of patients were sensitized to complex patterns that incorporated Blot 5, Gly d 2, and/or Lep d 2, underscoring the broader allergenic exposure in these regions.

Most patients were sensitized to multiple HDM allergen components. More than half of the individuals (52%) were sensitized to between 6 and 11 HDM components. Marked regional disparities emerged: in Valencia, nearly half of the patients (44%) were sensitized to no more than 5 allergens, and none exceeded 11. Conversely, in the Canary Islands, more than half of the patients (52.8%) exhibited sensitization to 11 to 13 allergens, with 86.8% reacting to at least 8 components, highlighting the high polysensitization burden in this environment.

Clinical outcome analyses identified significant associations between sensitization to specific molecular allergens and disease phenotypes. Fig E2 (in the Online Repository available at www.jaci-global.org) summarizes findings for AR (A) and asthma (B) in the total population. Regarding AR, sensitization to Tyr p 2 was linked to persistent forms of the disease (POR: 2.33; 95% CI 1.19-4.60). In region-specific analyses (data not shown), the strongest association was observed in the Basque Country (POR: 4.71; 95% CI 1.02-21.78). Interestingly, Lep d 2 demonstrated a divergent role: in Galicia, it was associated with a lower risk of moderate-to-severe AR (POR < 1), whereas in the Basque Country, its presence correlated with an increased risk of severe AR (POR: 5.02; 95% CI 1.07-23.60).

Regarding asthma, sensitization to Blot 5 showed a moderate association with disease risk (POR: 3.64; 95% CI 2.23-5.94), along with core HDM components such as Der f 1, Der f 2, Der p 1, Der p 2, and other minor allergens (Der p 21, Der p 5, Der p 7, Gly d 2, Blot 21, and Blot 10). Regional analyses (data not shown) reinforced these findings: in Galicia, asthma risk was particularly elevated in patients sensitized to Der f 2 (POR: 4.71; 95% CI 1.54-14.39), Der p 2 (POR: 3.92; 95% CI 1.41-10.92), and Der p 1 (POR: 3.49; 95% CI 1.49-8.21). Sensitization to Lep d2 was associated with persistent asthma in the Canary Islands (POR: 3.87; 95% CI 1.94-7.73), whereas in Galicia, Tyr p 2 also showed a strong association with persistent asthma (POR: 3.77; 95% CI 1.27-11.24). In the Basque Country, Der p 7 showed the strongest association with persistent asthma (POR: 5.71; 95% CI 1.33-24.62), suggesting that certain molecular allergens could serve as biomarkers for more severe or difficult-to-control asthma phenotypes.

## Discussion

This study provides the most comprehensive molecular characterization of HDM sensitization patterns in Spain to date, encompassing 5 regions that represent distinct geographic and climatic conditions, all characterized by high relative humidity and intense environmental mite exposure. The findings confirm that Der p 23, Der f 2, Der p 2, Der p 1, and Der f 1 are the major allergens in the Spanish population, consistent with prior reports from other European cohorts in which group 1 and 2 allergens predominate.^6^, ^7^, ^8^ The high prevalence of Der p 23 observed in our population further reinforces its growing recognition as a clinically relevant component, particularly given its association with more severe allergic phenotypes and poorer asthma control described in previous studies.^9^^,^^10^ Der p 23 showed high prevalence in our cohort, although its association with clinical severity was not demonstrated in this study.

One of the most notable contributions of this study is the demonstration of significant regional variability in sensitization profiles, which likely reflects the interplay between environmental conditions and allergen exposure intensity. Although all selected regions share a common feature of high ambient humidity, differences in climate, housing conditions, and lifestyle may explain the heterogeneity observed. The Canary Islands, for instance, exhibited the highest polysensitization rates, with patients commonly reacting to Der p 5, Der p 7, Der p 21, and Blot 5 in addition to the canonical allergens. These findings mirror patterns reported in tropical and subtropical environments, where Blomia tropicalis plays an important role in the allergic sensitization landscape. By contrast, patients from Valencia showed the lowest serodominance per individual, a finding that could be related to comparatively lower humidity variation and potentially different exposure dynamics, despite the coastal influence.

Analysis of patients’ sensitization profiles adds an additional level of clinical relevance. More than half of the cohort was sensitized to 6 or more HDM components, but this burden was unevenly distributed across regions. In the Canary Islands, where over half of the patients were sensitized to 11 to 13 allergens, the complexity of the molecular profile poses significant challenges for diagnosis and treatment, suggesting that standard extracts might fail to provide adequate coverage in such contexts. Conversely, in Valencia, nearly half of the patients reacted to only 1 to 5 allergens, reinforcing the need for region-specific strategies when planning AIT.

Beyond descriptive patterns, the clinical associations uncovered in this study highlight the potential of CRD as a tool for risk stratification. Sensitization to Blot 5 was associated with a moderate, but significant increase in asthma risk, an observation that aligns with previous findings in populations exposed to *Blomia* species.^11^^,^^12^ Moreover, Tyr p 2 and Lep d 2 exhibited region-dependent associations with disease severity: Lep d 2 appeared to reduce the likelihood of moderate or severe rhinitis in Galicia while significantly increasing it in the Basque Country, a paradox that may reflect differences in exposure context or sample size limitations. Similarly, sensitization to Tyr p 2 was strongly linked to persistent rhinitis and asthma, particularly in Galicia, whereas Der p 7 was the most relevant marker for persistent asthma in the Basque Country. These results suggest that certain molecular allergens could serve as biomarkers for severe or difficult-to-control disease, opening avenues for personalized treatment approaches. Although component-resolved diagnostics provide additional molecular detail, our study does not directly compare CRD with extract-based testing. Therefore, CRD should be considered complementary rather than superior in routine clinical practice.

When compared with previous studies, our findings are consistent in confirming the dominance of group 1 and 2 allergens and the relevance of Der p 23, but they also reveal distinctive features of the Spanish population, particularly the strong contribution of storage mites and *Blomia* in insular regions.^9^^,^^12^ Future patient-level studies should evaluate Der p 23 monosensitization phenotypes. These differences are likely driven by environmental and housing conditions that favor a broader and more sustained exposure to multiple mite species. From a clinical perspective, this diversity reinforces the need to move beyond extract-based diagnostics, which cannot capture such complexity, and adopt CRD as a standard tool for identifying high-risk profiles and selecting the most appropriate AIT formulations.

The implications of these findings are highly relevant for clinical practice. These findings suggest that regional exposure patterns should be considered when selecting allergen immunotherapy formulations. Furthermore, identifying patients sensitized to components associated with more severe phenotypes could enable earlier intervention, optimized therapy, and better disease control. In this context, the ALEX^2^ platform, used in the present study, demonstrated its value as a comprehensive tool capable of simultaneously assessing a wide spectrum of allergens with high analytical performance.^13^, ^14^, ^15^

This study has several strengths, including its multicenter design, the use of a state-of-the-art molecular diagnostic platform, and the inclusion of regions with contrasting environmental conditions, which allowed for a robust assessment of geographic variability. Nonetheless, certain limitations should be acknowledged. The cross-sectional design precludes causal inference, and clinical phenotypes were assessed at a single time point, limiting the ability to evaluate longitudinal outcomes. Sample size differences across regions could also influence the power to detect some associations, particularly for less frequent allergens. Because participants were recruited from specialized allergy clinics, the prevalence of asthma and polysensitization likely reflects referral bias and may not represent the general population. Future studies should aim to validate these findings in larger cohorts, ideally incorporating longitudinal follow-up to assess the predictive value of molecular sensitization patterns for disease progression and response to immunotherapy.

In conclusion, this study confirms the central role of Der p 23, Der f 2, Der p 2, Der p 1, and Der f 1 as major HDM allergens in Spain, although highlighting substantial regional variability and the clinical relevance of less common components such as Blot 5, Lep d 2, and Tyr p 2. These results emphasize the importance of CRD for accurate diagnosis, patient stratification, and personalized AIT strategies, particularly in regions with high allergenic pressure and complex exposure patterns. Incorporating molecular diagnostics into routine practice has the potential to improve treatment outcomes and reduce the burden of HDM-related allergic diseases in Spain.

## Disclosure statement

DIATER Laboratories has funded the serological tests to contribute to increase the knowledge of mite serodominance in Spain.

Disclosure of potential conflict of interest: The authors declare that they have no relevant conflicts of interest.

Declaration of AI and AI-assisted technologies in the writing process: The authors used ChatGPT to revise the language and adapt the manuscript to the journal’s style. After using this tool, the authors reviewed and edited the content.

## Figures and Tables

**Supplementary Figure S1:**
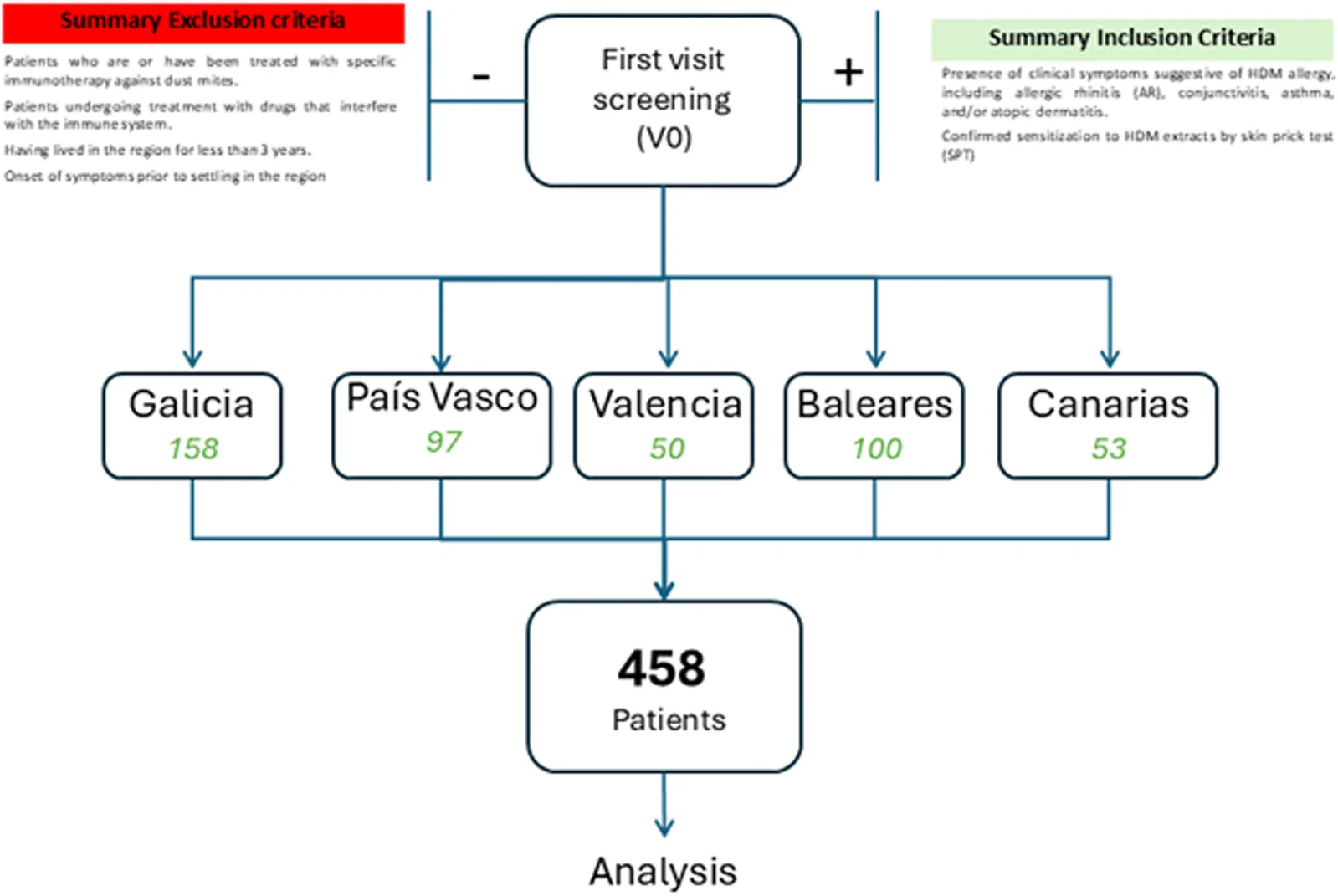


**Supplementary Figure S2:**